# Mechanisms of Attenuation of Pulmonary V’O_2_ Slow Component in Humans after Prolonged Endurance Training

**DOI:** 10.1371/journal.pone.0154135

**Published:** 2016-04-22

**Authors:** Jerzy A. Zoladz, Joanna Majerczak, Bruno Grassi, Zbigniew Szkutnik, Michał Korostyński, Sławomir Gołda, Marcin Grandys, Wiesława Jarmuszkiewicz, Wincenty Kilarski, Janusz Karasinski, Bernard Korzeniewski

**Affiliations:** 1 Department of Muscle Physiology, Chair of Physiology and Biochemistry, Faculty of Rehabilitation, University School of Physical Education, Krakow, Poland; 2 Dipartimento di Scienze Mediche e Biologiche, Università degli Studi di Udine, Udine, Italy; 3 Faculty of Applied Mathematics, AGH-University of Science and Technology, Krakow, Poland; 4 Department of Molecular Neuropharmacology, Institute of Pharmacology, Polish Academy of Sciences, Krakow, Poland; 5 Department of Bioenergetics, Institute of Molecular Biology and Biotechnology, Adam Mickiewicz University, Poznan, Poland; 6 Department of Cell Biology and Imaging, Institute of Zoology, Jagiellonian University, Krakow, Poland; 7 Faculty of Biochemistry, Biophysics and Biotechnology, Jagiellonian University, Krakow, Poland; Norwegian University of Science and Technology, NORWAY

## Abstract

In this study we have examined the effect of prolonged endurance training program on the pulmonary oxygen uptake (V’O_2_) kinetics during heavy-intensity cycling-exercise and its impact on maximal cycling and running performance. Twelve healthy, physically active men (mean±SD: age 22.33±1.44 years, V’O_2peak_ 3198±458 mL ∙ min^-1^) performed an endurance training composed mainly of moderate-intensity cycling, lasting 20 weeks. Training resulted in a decrease (by ~5%, *P* = 0.027) in V’O_2_ during prior low-intensity exercise (20 W) and in shortening of τ_p_ of the V’O_2_ on-kinetics (30.1±5.9 s vs. 25.4±1.5 s, *P* = 0.007) during subsequent heavy-intensity cycling. This was accompanied by a decrease of the slow component of V’O_2_ on-kinetics by 49% (*P* = 0.001) and a decrease in the end-exercise V’O_2_ by ~5% (*P* = 0.005). An increase (*P* = 0.02) in the vascular endothelial growth factor receptor 2 mRNA level and a tendency (*P* = 0.06) to higher capillary-to-fiber ratio in the vastus lateralis muscle were found after training (n = 11). No significant effect of training on the V’O_2peak_ was found (*P* = 0.12). However, the power output reached at the lactate threshold increased by 19% (*P* = 0.01). The power output obtained at the V’O_2peak_ increased by 14% (*P* = 0.003) and the time of 1,500-m performance decreased by 5% (*P* = 0.001). Computer modeling of the skeletal muscle bioenergetic system suggests that the training-induced decrease in the slow component of V’O_2_ on-kinetics found in the present study is mainly caused by two factors: an intensification of the each-step activation (ESA) of oxidative phosphorylation (OXPHOS) complexes after training and decrease in the ‘‘additional” ATP usage rising gradually during heavy-intensity exercise.

## Introduction

During rest to moderate-intensity exercise transition (i.e. below the lactate threshold–(LT)) pulmonary oxygen uptake reaches a steady state within about 2–3 minutes (see e.g. [1]). However, in case of heavy-intensity exercise, accompanied by plasma lactate accumulation [2] a progressive increase in V’O_2_ is observed—known as the slow component of the V’O_2_ on-kinetics [1,3,4]. This “excess” in oxygen uptake originates at the LT [4,5] but becomes progressive after the so called “critical power” [3,6,7]. The critical power represents the maximal power output generated by the working muscles above which no steady state in muscle and pulmonary V’O_2_ and blood pH and lactate concentration can be reached during sustained bouts of exercise. This is accompanied by a progressive decrease in muscle PCr and a progressive accumulation of muscle metabolites involved in muscle fatigue such as P_i_, H^+^, ADP_free_ and others (for details see, e.g. Refs. [3,6,7]).

It has been reported by Poole et al. [8] that most of the amplitude (> 80%) of the slow component of the pulmonary V’O_2_-on kinetics in humans is generated in the working muscles. This finding is further supported by more recent experimental data by Zoladz et al. [9] showing a V’O_2_ slow component-like response (i.e. a progressive increase in V’O_2_: force ratio) in electrically stimulated fatiguing isolated dog gastrocnemius muscle preparation *in situ*. The magnitude of this response was similar to the magnitude of the slow component of V’O_2_ on-kinetics observed in humans [9]. Therefore, the underlining mechanism of the pulmonary slow component of the V’O_2_ on-kinetics seems to operate mostly within the working skeletal muscles.

It has been demonstrated that a few weeks of endurance training decreases the amplitude of the pulmonary V’O_2_ slow component in humans [10,11,12] and the magnitude of the non-linear increase in the V’O_2_ -power output relationship in humans [13], leading to an increase in power generating capabilities at V’O_2max_ despite of unchanged V’O_2max_ after the training (V’O_2max_). However, the physiological mechanism responsible for the training-induced attenuation of the slow component of the V’O_2_ on-kinetics remains unclear.

The slow component of V’O_2_ kinetics occurring in the working muscle can be potentially caused by an efficiency decrease on the side of ATP production (decrease in the P/O ratio) and/or on the side of ATP consumption (increase in the ATP usage/power output ratio) [1,3,14]. It was proposed in a recent experimental study by Cannon et al. [15] that both the above mentioned factors can be involved.

Since the pioneer studies by Holloszy and coworkers showing the training-induced increase of mitochondrial enzymes activities [16], an improvement of muscle performance has been associated with a decrease of metabolites changes during exercise due to an enhanced mitochondrial biogenesis [17,18]. There are, however, experimental evidences showing that the training-induced enhancement of muscle metabolic stability can precede mitochondrial biogenesis in skeletal muscles [19,20]. Zoladz et al. [20] postulated that this effect could be achieved by the training-induced intensification of each-step activation (ESA) in skeletal muscles (this effect of training-induced ESA increase was first proposed in a theoretical study by Korzeniewski and Zoladz [21]). According to the ESA mechanism, an increase of ATP usage during exercise is accompanied by a direct simultaneous activation of all oxidative phosphorylation complexes (complex I, III, IV, ATP synthase, ATP/ADP carrier, P_i_ carrier), NADH-supply metabolic block and glycolysis, probably by some cytosolic Ca^2+^-related mechanism (involving e.g., protein phosphorylation), allowing to maintain relatively stable concentrations of ATP, ADP_free_, PCr, P_i_ and NADH in the working muscle while greatly increasing the turnover of those intermediates [22,23,24]. In general, the ESA concept is similar to the simultaneous activation concept proposed in relation to skeletal muscle by Hochachka and co-workers [25], although these authors did not specify which and how many enzymes would be activated (therefore the version of parallel activation proposed by Korzeniewski is called ‘‘each-step activation” in order to avoid confusion), and denied the possible role of Ca^2+^. It is worth noting that this concept of maintaining energy balance in the muscle during its contractions has recently received a significant experimental support (see e.g. Refs. [20,26,27,28]).

In a recent theoretical study [29], carried out using a computer model of skeletal muscle bioenergetic system developed previously [30,31], it was proposed that the slow component of the V’O_2_ on-kinetics can be caused by inhibition of ATP production by anaerobic glycolysis by progressive cytosol acidification (together with a slow decrease in ATP supply by creatine kinase). On the other hand, cytosol acidification, through glycolysis inhibition, slows down its further progress (this is a self-limiting mechanism). Moreover, the study [29] suggested also a significant contribution to the slow component of a gradual increase of ATP usage during exercise of constant power output (‘‘additional” ATP usage) [1,9,15,32]. Therefore, training-induced attenuation of either metabolic acidosis or ‘‘additional” ATP usage, or both during exercise of heavy-intensity should decrease the magnitude of the slow component [29].

The main aim of the present study was to evaluate the effect of prolonged endurance training composed mainly of moderate-intensity exercise on the V’O_2_ on-kinetics in young healthy men in relation to changes in some potential factors affecting the muscle metabolic stability like: mitochondrial biogenesis (including OXPHOS activity), changes in proportion of various myosin heavy chains (MyHCs) content and appearance of markers of muscle capillarization. Finally, using a computer model of oxidative phosphorylation we have examined the importance of the training-induced enhancement of muscle metabolic and pH stability caused by an enhancement of ESA and by a decrease in the ‘‘additional” ATP usage during exercise, on the training-induced attenuation of the slow component of the V’O_2_ on-kinetics. We have hypothesized that the training-induced attenuation of the slow component of the V’O_2_ on-kinetics will be accompanied by enhancement of muscle metabolic stability caused mainly by the training-induced increase of ESA and decrease in the ‘‘additional” ATP usage during exercise, as well as by a lower cytosol acidification caused by a smaller (anaerobic) glycolysis activation (through direct activation and ADP increase). Moreover, we have hypothesized that the training-induced attenuation of the slow component of V’O_2_ on-kinetics will result in enhancement of physical performance during high-intensity exercise, despite an unchanged peak oxygen uptake (V’O_2 peak_).

## Materials and Methods

### Subjects

Twelve untrained, but physically active male volunteers (mean ± SD: age: 22.3 ± 1.44 years, body weight 76.84 ± 14.4 kg, height 180.3 ± 7.89 cm, BMI 23.6 ± 3.8 kg · m^2^, V’O_2max_ 3198 ± 458 mL · min^-1^), took part in this study after giving informed written consent. The recruitment of subjects to this study was based on public announcement to the student society of our University the possibility to participate in this study, by those who meet the following criteria: male subjects, aged 20–25 years old, no involvement in professional training, no smoking status, no alcohol/drugs addiction, good general health. From the volunteers we have excluded those who did not pass medical examinations based on general medical check-up, blood testing and ECG recording. In a questionnaire, performed prior to the study, the subjects declared that they neither participated in a professional training programs in the past, nor do so currently, but they did perform various kinds of physical activities on a recreational level lasting on average 3 hours per week.

The study was conducted with permission of the Ethical Committee (Commission for Bioethics at the Regional Medical Chamber in Krakow, opinion Nr 48/KBL/OIL/2009) according to principles established in the Declaration of Helsinki for research on human subjects.

### Exercise protocols

All subjects performed two types of exercise protocols before and after training: an incremental exercise until exhaustion and a constant power output of heavy-intensity exercise (see below). All exercise tests were performed on a cycloergometer Ergo-Line GmbH & Co KG 800s (Bitz, Germany) with computer controlled power outputs. The pedaling rate during all tests was 60 rev · min^-1^. Subjects were asked to avoid heavy exercise the day before and the day of the tests as well as to avoid caffeinated and alcoholic beverages 24 hours before the tests.

#### Incremental exercise

The incremental test started with a 6 minutes rest, i.e. with the subject sitting on the cycloergometer, followed by a gradual increase of power output by 30 W every 3 minutes until voluntary exhaustion—maximal power output (PO_max_) [5]. The incremental test was performed two days before and two days after the twenty-weeks of endurance training (see below).

#### Constant power output test

The high intensity constant power output test consisted of 6 minutes of rest sitting on the cycloergometer, followed by 6 minutes prior low-intensity exercise (cycling at 20 W) (baseline) and 6 minutes heavy-intensity exercise cycling at the power output corresponding to 50% Δ. The power output corresponding to 50% Δ was calculated as the difference between the power output reached at V’O_2peak_ and the power output obtained at the LT [50% Δ = PO_LT_ + 0.5 (PO_max_−PO_LT_)]. The level of the gas exchange variables determined during cycling at 20 W before the main work load was used as a baseline for determining the V’O_2_ on-kinetics. In order to improve the signal to noise ratio, the 6-minutes bouts of cycling exercise were carried out three times. The heavy-intensity bouts of exercise were performed on separate days.

### Endurance training program

The volunteers underwent a supervised endurance training program on a cycle ergometer (Monark 874 E, Monark Exercise AB, Vansbro, Sweden), four times a week for 20 weeks. Training consisted of two different exercise protocols (each lasting 40 minutes): a moderate-intensity continuous cycling (CC) and a high-intensity intermittent cycling (IC). CC was performed at the power output corresponding to 90% of the previously determined lactate threshold (90% LT), whereas IC consisted of 6 minutes of unloaded cycling followed by a 3 minutes exercise bout at the power output corresponding to 50% Δ, repeated four times and finishing with 4 minutes of unloading cycling. The power output corresponding to 50% Δ was calculated as the difference between the power output reached at V’O_2peak_ and the power output obtained at the LT [50% Δ = PO_LT_ + 0.5 (PO_max_−PO_LT_)]. To maintain an adequate training stimulus, the power output was adjusted after the first 5 weeks of training and subsequently after each of the five weeks periods of the study. From the beginning of the 6^th^ week until the end of the 10^th^ week, the unloaded cycling in the IC sessions was replaced by cycling at the 90% LT. During the next 5 weeks, the power output corresponding to 90% LT was increased by 5% both in CC and IC protocols (from week 11^th^ to 15^th^), and by 15% during the last five weeks of the training program (from week 16^th^ to 20^th^). The CC was performed on Tuesdays and Fridays, and IC on Mondays and Thursdays. Each training session was monitored using a heart rate monitor (Polar S810, Polar Electro Oy, Kempele, Finland) and supervised by one of the authors (for details see Ref. [28]). The endurance-training program applied in the present study was composed mainly of moderate-intensity cycling since ∼86% of its total duration was performed below LT; i.e., at ∼50% of V’O_2peak_, and only ∼14% of its total duration was performed in heavy-intensity domain at ∼75% of V’O_2peak_. During the training period subjects were asked to keep their normal mixed diet and to avoid taking any nutritional supplements or consuming alcohol.

### Measurements

#### Gas exchange variables

Gas exchange variables were recorded continuously breath-by-breath using the Oxycon Champion, Mijnhardt BV (Bunnik, The Netherlands). Before each test the gas analyzers were calibrated with certified calibration gases as previously described [5]. During the incremental exercise test a medium size Rudolph mask was used (dead space 90 mL), whereas during the constant power output tests the subjects were breathing via a small sized mouthpiece (dead space 40 mL).

#### Heart rate (HR)

Heart rate was determined from the ECG tracing, registered continuously by the Hellige SMS 181 unit (Germany).

#### Blood sampling

Blood samples were collected via an Abbot Int-Catheter, Ireland (18G/1.2 × 45 mm) inserted into an antecubital vein and connected to an extension set using a “T” Adapter (SL Abbot, Ireland) (the length of the tube was 10 cm). Blood samples (0.5 mL each) were taken at rest– 6 minutes before the test and at the end of each step of the incremental test (10 s before the end of each step) as well as at the end of the incremental protocol. Samples were transferred to 1.8 mL Eppendorf tubes containing 1 mg ammonium oxalate and 5 mg sodium fluoride, mixed for about 20 seconds and centrifuged.

#### Plasma lactate concentration [La^-^]_pl_

The supernatants containing blood plasma (about 0.2 mL) were stored at minus 40°C until further analysis of lactate concentration ([La^-^]_pl_), which was determined using an automatic analyzer (Vitros 250 Dry Chemistry System, Kodak, Rochester, NY, USA). Detection limit was 0.5 mmol · L^-1^.

#### Lactate threshold (LT)

The lactate threshold (LT) was identified, as previously described [5], as the highest power output above which [La]_pl_ showed a sustained increase of > 0.5 mmol · L^-1^ · step ^-1^.

#### Running performance

Running performance at 1,500-m was determined during a competitive race performed by the subjects on a typical track and field artificial track (tartan) before and after endurance training. Running capacity was evaluated by determining the running velocity at 85% HR_max_ (v at 85% HR_max_); this was carried out on the basis of the linear relationship between running velocity and HR, established for each subject during a running field test, as described previously by Zoladz et al. [33].

#### Muscle biopsy and analyses

Muscle biopsies were obtained under local anesthesia (1% Lignocainum Hydrochloricum, Polfa, Warszawa, Poland) from the right *vastus lateralis m*. *quadricipitis femoris* approximately 15 cm above the upper margin of *patella* by utilizing a 2 mm ø biopsy needle (Pro-Mag^™^ I 2.2, Angiotech, Vancouver, Canada). The sufficient size of muscle samples for further measurements in this study were obtained from 11 out of 12 studied subjects. The specimens were frozen immediately in liquid nitrogen and used for further measurements of gene expression, Western immunoblotting and muscle capillarization. Muscle biopsies were taken before and after the endurance training program. The second muscle biopsy (i.e. after training) was obtained 74.0 ± 2.8 hours after the last training session.

#### RNA isolation and quantitative PCR

The muscle biopsies specimens frozen immediately in liquid nitrogen were used for measuring abundance levels of selected transcripts. Frozen tissue samples were homogenized in 1 mL Trizol reagent (Invitrogen, Carlsbad, CA, USA) and total RNA was extracted according to the manufacturer protocol. RNA concentration was measured using a ND-1000 Spectrometer (NanoDrop Technologies Inc., Montchanin, DE, USA). Reverse transcription was performed with Omniscript Reverse Transcriptase enzyme (Qiagen Inc., Valencia, CA, USA) at 37°C for 60 minutes. The qPCR reactions were performed using Assay-On-Demand TaqMan probes (*VEGFA*, Hs00900055_m1; *FLT1*, Hs01052961_m1; *KDR*, Hs 00911700_m1; *HIF1A*, Hs00153153_m1; *MFN1*, Hs00966851_m1; *MFN2*, Hs00208382_m1, *OPA1*, Hs01047018_m1) according to the manufacturer's protocol (Applied Biosystems, Foster City, CA, USA) and were run on the CFX96 Real-Time system (Bio-Rad, Foster City, CA, USA). Expression of the hypoxanthine-guanine phosphoribosyltransferase (*HPRT1*, Hs01003270_g1) transcript with stable levels following experimental conditions was quantified to control for variation in cDNA amounts. The abundance of RNA was calculated as 2^-(threshold cycle)^. Data were normalized to the expression levels of the HPRT1 mRNA.

#### Muscle protein contents

Protein extraction was performed using ProteoExtract^™^ Complete Mammalian Proteome Extraction kit (Calbiochem No. 539779, Millipore, Merck KGaA, Darmstadt, Germany) designed to extract total proteins from mammalian tissue. Protein concentrations in skeletal muscle extracts were measured spectrofluorometrically using Qubit^™^ Fluorometer (Invitrogen, Life Technologies, Carlsbad, CA, USA). Skeletal muscle tissue extracts were stored at -80°C until further measurements.

#### Western blotting

Skeletal muscle tissue protein extracts were mixed with sample buffer (BioRad, #161–0737) with addition of 2.5% 2-mercaptoethanol and were denatured at 95°C for 5 min. Gels (4% stacking and 12% separating gels or Mini Protean TGXT 4–15% cat. No 456–1083, BioRad) were loaded with denatured extracts i.e. 11 μg of total protein in case of sarco(endoplasmic)reticulum Ca^2+^ ATPase 1 (SERCA1), sarco(endoplasmic)reticulum Ca^2+^ ATPase 2 (SERCA2), glyceraldehyde 3-phosphate dehydrogenase (GAPDH) and 7 μg of protein in case of myosin heavy chain type 2 (MyHC2). After this step electrophoresis proteins were transferred overnight at a constant voltage (30 V) at 4°C in transfer buffer to nitrocellulose membranes (Amersham Hybond^™^, GE^™^ Healthcare, Pittsburgh, PA, USA). The membranes were then incubated in 5% milk in Tris-buffered saline and 0.1% Tween 20 (TBST) for 12 hours at 4°C. Membranes were subsequently incubated for 1 hour, at room temperature in primary antibodies specific to: SERCA1 (ab2819, Abcam, Cambridge, UK), diluted 1:2500 in 2% BSA in Tris-buffered saline and 0.1% Tween 20 (TBST); SERCA2 (ab2861, Abcam, Cambridge, UK), diluted 1:1000 in 2% BSA in TBST; GAPDH (ab8245, Abcam, Cambridge, UK) diluted 1:5000 in 2% BSA in TBST; MyHC2 (ab51263, Abcam, Cambridge, UK) diluted 1:1000 in 2% BSA in TBST. After incubation membranes were washed in TBST 3 x 10 minutes and then incubated in secondary antibody (rabbit anti-mouse IgG H&L (HRP), ab97046, Abcam, Cambridge, UK) diluted 1:30 000 in 2% BSA in TBST for 1 hour, at room temperature. After that membranes were washed in TBST 3 x 10 minutes. For the detection of SERCA1, SERCA2, GAPDH, MyHC2, membranes were developed with chemiluminescent detection (ECL Western Clarity, #170–5060, BioRad, Hercules, CA, USA) in GeneGnome 5 (GeneSys 1.2.7.0, Syngene Bio Imaging, Cambridge, UK). GeneTools Syngene (Cambridge, UK) analysis software was used for densytometric analysis. The values of SERCA1 and SERCA2 were normalized by reference to GAPDH and presented in arbitrary units (AU). The values of MyHC2 were normalized by reference to α-actin, and are presented in arbitrary units (AU).

#### Muscle morphometry

Muscle biopsy samples were prepared routinely for examination by electron microscopy. Small fragments of biopsies were fixed at 4°C in 6.25% glutaraldehyde solution in 0.1M sodium cacodylate buffer (pH 7.4; 1100 osmol) for 2 hours. Then they were cut into a few millimeters size blocks and washed for several hours in sodium cacodylate buffer (pH 7.4) containing sucrose to rise osmolality to 1100 osmol. The fixed and washed samples were then post fixed in 1% osmium tetroxide for 2 hours, dehydrated in increasing series of ethanol (70–100%) and embedded in Epoxy resin (Epon 812). Polymerized blocks of tissues were cut on Reichert Ultracut microtome 701 701 (Reichert-Jung, Wien, Austria) approximately at right angles to the axis of the muscle fibers. The semi thin sections (1–0.5 μm) were stained in the mixture of 1% toluidine and methylene blue (1:1) and photographed in the Nikon OPTIPHOT-2 (NIKON, Tokyo, Japan) light microscope, with the aid of × 20 objective photographed by NIKON Digital Camera DXM 1200F (NIKON, Tokyo, Japan), and stored in a computer’s memory.

**Fig 1:**
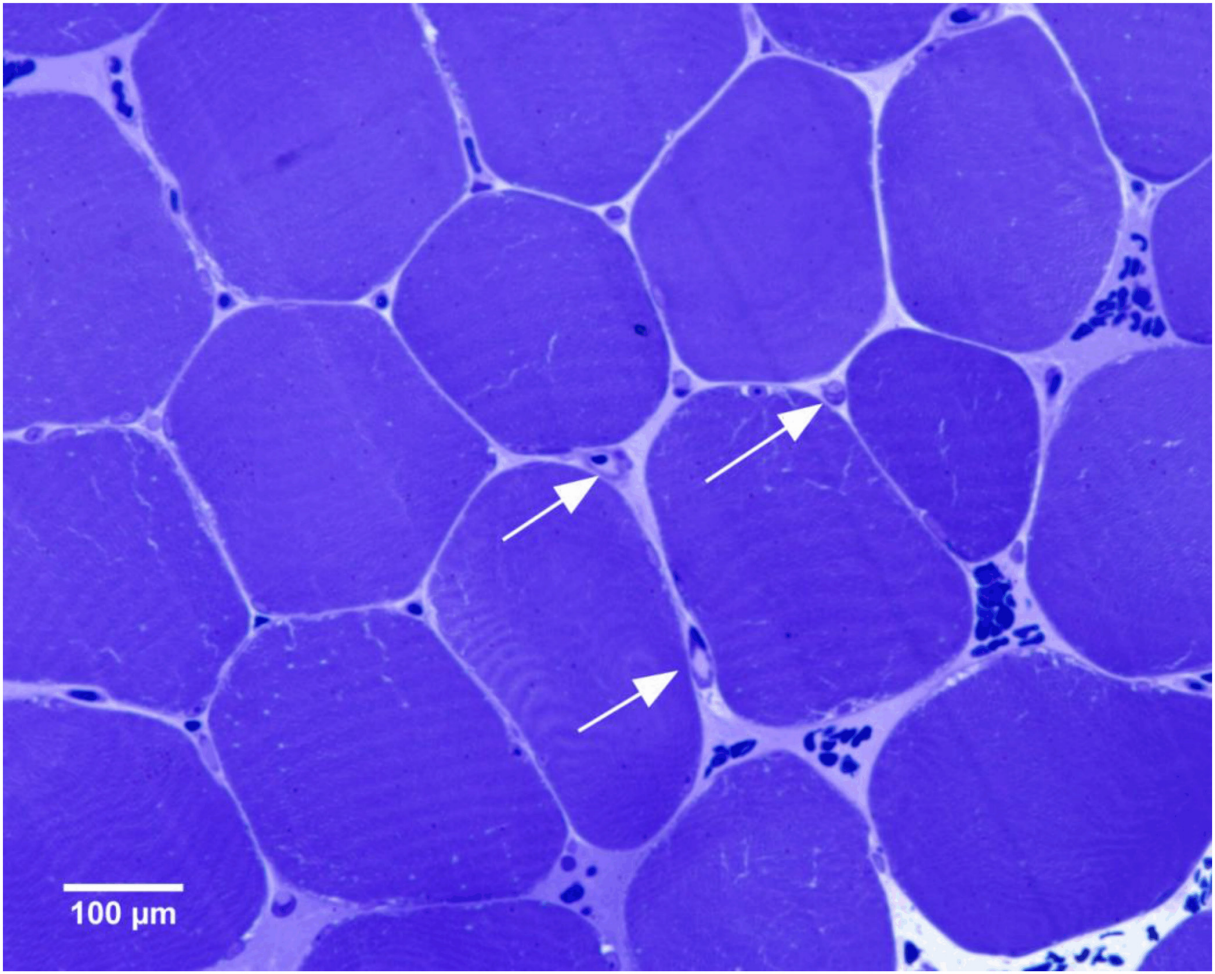
Transverse section of human muscle fibers, preserved in epoxy resin. A representative area of a part of vastus lateralis muscle stained with the mixture of toluidine and methylene blue is showed. The arrows indicate the cross section of capillaries.

The calculation of muscle fibres size and number of capillaries were performed directly from the pictures on the computer screen (see Fig 1) on which the test grid was overlaid using *Image J* 1.40 g program (Wayne Rosland N.I.H., USA, http//rsb.infonihgov/ij/Java 1.60–05) [34]. The grid constant was calibrated for the magnification used with a stage micrometer (1/100 mm, Carl Zeiss, Oberkochen, Germany). The constant test grid used covers the whole section and had 48 squares, what was established to have 900 μm^2^ each. The capillary numbers were related to the muscle fibre numbers as a reference space in all samples to avoid variation due to the unreliable preservation of the intercellular spaces by the preparation procedures. Therefore size of the area used to estimate the muscle fibre size and capillary number was 43,200 μm^2^.

The capillary network of all striated muscles consists of capillaries which lie parallel and transversal to the fibres. In general the density of capillary network is consequently determined by counting capillary cross-sections on muscle cross-sections. In the semi-thin sections stained with the mixture of toluidine and methylene blue, the capillary cross section was easily discernible. Structures which could not be identified unequivocally as capillaries were not included in the evaluation. The muscle fibre sizes were expressed as the area of their profiles.

#### Modelling and estimation of V’O_2_ kinetic

**Fig 2:**
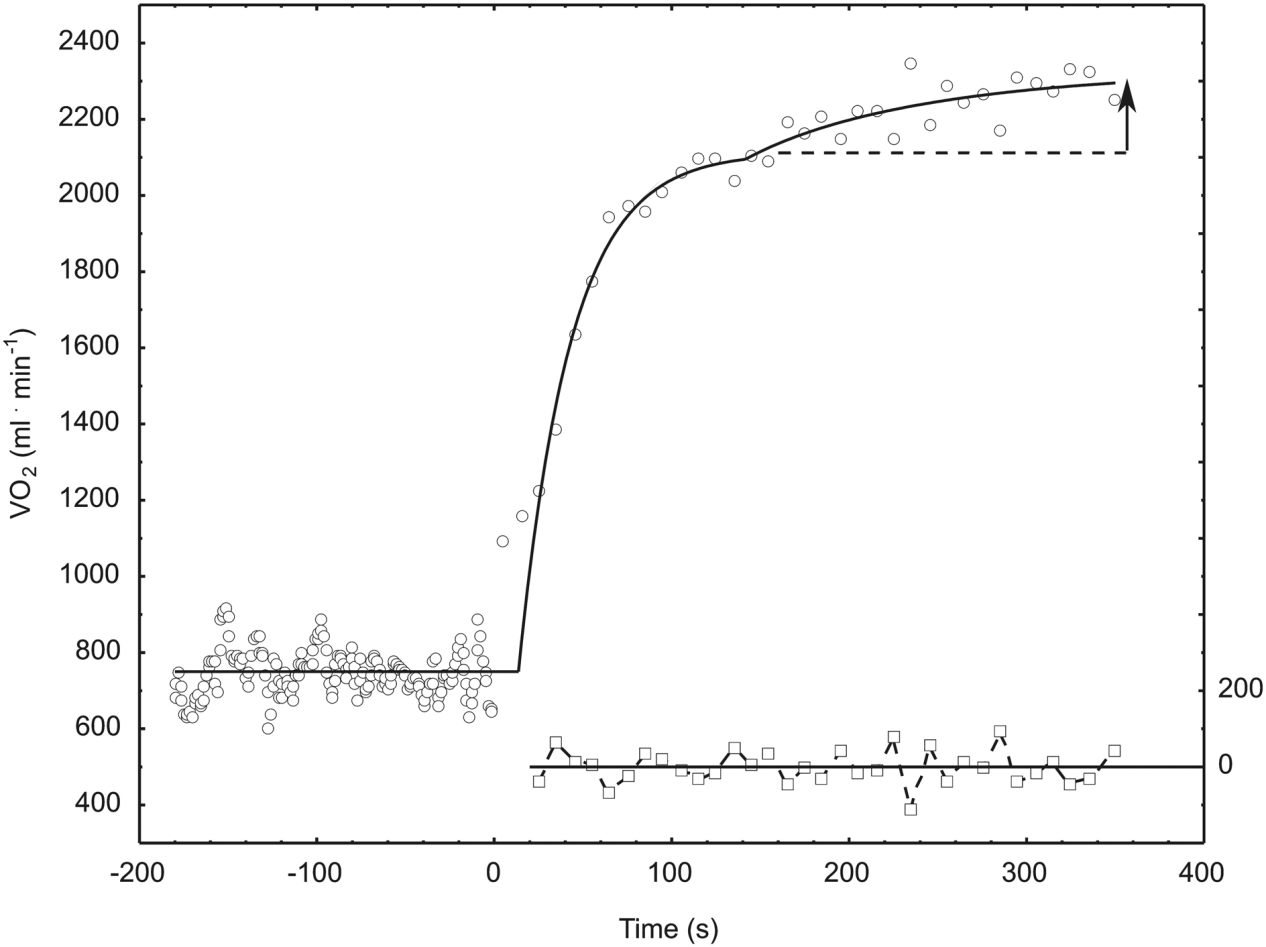
Models fitted to subject 3 data collected during baseline-heavy-intensity exercise transition before training and the corresponding residuals. The arrow represents the size of the slow component, which is defined as the difference between the value reached at the end of the exercise, and the value of the first (fast) exponential component (represented with the broken line).

The raw breath-by-breath data were preprocessed to remove occasional, artifactual values resulting from, e.g., swallowing or coughing, by means of a procedure similar to that described by Lamarra et al. [35]. V’O_2_ responses from the bouts of repeated exercises were time-aligned to the onset of the exercise and interpolated on a second-by-second basis. The resulting data were then, for each subject, superimposed, averaged over the transitions of the exercise and, finally, averaged over consecutive 10 s time intervals, as in, e.g., Ref. [36], in order to reduce the noise. *t* = 0 was set at the onset of exercise. The first cardio-dynamic phase of the response was not modelled. Correspondingly, the data from the first 20 s were ignored (see Fig 2).

The V’O_2_ responses were modelled with a double-exponential curve

$$V^{\prime }O_{2}(t)\;=\;V^{\prime }O_{2\;}+A_{1}\times (l-e^{-(t-TD_{1})/\tau_{1}})+A_{2\;}\times (l-e^{-(t-TD_{2})/\tau_{2}})$$

(see e.g., Refs. [37,38]). V’O_2_ (0) is the baseline value, calculated as the average over the 3 minutes before the onset of the exercise. The first exponential term starts at *t* = TD_1_ and the second one, called the slow component, starts at *t* = TD_2_ > TD_1_.

In all cases, the relevant models were fitted to the corresponding data by the least-squares method, using the *Nonlinear Estimation* module of STATISTICA, version 6. Several starting points for the iterative minimization procedure were tried, in order to find the best fit.

The value of the slow component was computed as the difference between the value reached at the end of the exercise, and the value of the first (fast) exponential component at the end of the exercise, i.e.,

$$Slow\;Comp\;=\;V^{\prime }O_{2}(360)-V^{\prime }O_{2}(0)-A_{1}\times (l-e^{-(360-{TD}_{1})/\tau_{1}})\approx V{\prime O}_{2}(360)-V\prime O_{2}(0)-A_{1}$$

### Statistics

The results are presented in Tables and Figures as means ± SD. Additionally, the Tables demonstrate 95% confidence intervals of the means (95% CI). The significance of the difference between τ_1_ (and other variables) measured before and after the training was tested with the matched-pairs Wilcoxon signed-rank test, which means that for each subject the change Δτ_1_ of τ_1_ was computed and the Wilcoxon signed-rank test was used to check whether those differences were significantly non-zero. Exact (i.e. non-asymptotic), two-sided *P*-values were computed, as described in Zoladz et al. [38].

The non-parametric test with the exact *P*-values was used because of the relatively small sample size (≤ 12 subjects), on which the comparisons were based. With 12 data points, it is not possible to reliably verify the assumption of normality, needed for an application of standard parametric tests, like the t-Student test. For the non-parametric Wilcoxon signed-rank test, one only needs to assume the symmetry of the distribution with respect to its median, which is much weaker and, in our opinion, acceptable for the present study.

### Computer simulations of muscle V’O_2_, metabolite changes and ATP fluxes during work transitions

In the present study we utilized the theoretical model of skeletal muscle cell bioenergetics, including anaerobic glycolysis, developed by Korzeniewski and Liguzinski [30], and based on an earlier model by Korzeniewski and Zoladz [31]. This model comprises oxidative phosphorylation (OXPHOS) complexes (complex I, complex III, complex IV, ATP synthase, ATP/ADP carrier, P_i_ carrier), aerobic and anaerobic glycolysis, creatine kinase (CK), ATP usage, NADH supply and proton efflux/influx to/from blood. The model comprises the inhibition of (anaerobic) glycolysis by protons [29,30].

The model has been broadly validated by comparison of its predictions with experimental data, and has been used in numerous theoretical studies (see e.g., Refs. [22,23,24]). The complete model description can be found on the web site: http://awe.mol.uj.edu.pl/~benio/.

The model (some of its parameters, in particular ATP usage activation in relation to rest during prior and main exercise, A_UT_, OXPHOS and NADH supply activation, A_OX_, and ‘‘additional” ATP usage after 6 min of main exercise) was (were) fitted to the experimentally-obtained V’O_2_ *vs*. time data obtained during the constant power output exercises. After this fitting, the model generated the V’O_2_ kinetics that reproduced well the observed V’O_2_ kinetics (see”Results”, “Computer simulations” section). Also other variable values, namely ADP, PCr, P_i_ and ATP concentrations, pH as well as ATP usage (UT), ATP supply by OXPHOS (OX), creatine kinase (CK) and anaerobic glycolysis (GL) were simulated and shown for illustration (see “Results”, “Computer simulations” section). The values of A_OX_ and A_GL_ also were fitted to give reasonable values of (changes in) ADP, PCr and pH (although they also co-determined the V’O_2_ kinetics).

In order to explain the difference in V’O_2_ for the same power output we assumed within the model, in order to fit experimental data for prior exercise, that the ATP cost of power output was by 5.5% greater in untrained muscle than in trained muscle both during the prior and main exercise. In other words, it was assumed that ATP usage for muscle contraction (by actomyosine-ATPase and Ca^2+^-ATPase) decreased by 5.5% at a given power output in the result of muscle training (for instance because of Ca^2+^ release from sarcoplasmic reticulum and SERCA down-regulation). This is an assumption made within the model in order to fit the experimental data. We assumed that the training-induced decrease in the pulmonary V’O_2_/P.O. ratio observed in experiment is a derivative of a decreases in the ATP/P.O. ratio and not in the O_2_/ATP ratio. This is because it was estimated previously [29] (based on the available data, see therein) that the proton leak, the main candidate for the factor affecting the O_2_/ATP ratio, is responsible for only about 1% of V’O_2_ during heavy exercise (although this issue requires further study), and therefore we assumed that a possible training-induced decrease in its intensity could not lower significantly the O_2_/ATP ratio. Consequently, we assumed (within the model, in order to fit experimental data) that during the low-intensity exercise (20 W) (baseline) ATP usage was directly activated A_UT_ = 20 times (in relation to rest) in trained muscle and A_UT_ = 20 * 1.055 = 21.1 times in untrained muscle. We assumed that during heavy-intensity exercise ATP usage was activated A_UT_ = 80 times (in relation to rest) in trained muscle and A_UT_ = 80 * 1.055 = 84.4 times in untrained muscle. In untrained muscle all OXPHOS complexes and NADH supply block were directly activated A_OX_ = A_UT_^0.35^ = 2.91 and 4.72 times in relation to rest during prior (low-intensity) and main (heavy) exercise, respectively (the power coefficient p = 0.35 is a measure of ESA intensity within the model). This represents the ESA mechanism [22,23,24] mentioned above. In trained muscle ESA was intensified, which means that OXPHOS complexes and NADH supply were activated A_OX_ = A_UT_^0.38^ = 3.12 and 5.29 times in relation to rest during prior (low-intensity) and main (heavy) exercise, respectively (again, the power coefficient p = 0.38 is a measure of ESA intensity). It was assumed that there was no significant anaerobic glycolysis during the low-intensity exercise (NADH supply by glycolysis + TCA cycle exactly matches NADH consumption by OXPHOS). During heavy-intensity exercise glycolysis was directly activated A_GL_ = A_UT_^0.48^ = 8.41 and 8.19 times in untrained and trained muscle, respectively (in this case p = 0.48 is a measure of parallel activation of glycolysis within the model). Therefore, the direct activation of glycolysis in general and anaerobic glycolysis in particular was higher in untrained than in trained muscle. Glycolysis was also more intensively activated indirectly in untrained muscle, through a greater increase in ADP. Both effects decreased the apparent gain in untrained muscles in relation to trained muscles. The need for a direct activation of glycolysis during rest-to-work transitions was demonstrated previously [30]. In the computer simulations the activation of the system related to the transition from the low-intensity exercise (20 W) to the heavy-intensity exercise was delayed by 24 s (0.4 min), with respect to the experimental data, in order to take into account the cardio-dynamic phase of the V’O_2_ on-kinetics. It was assumed, in order to fit the measured V’O_2_ kinetics, that in untrained muscle the ‘‘additional” ATP usage during heavy-intensity exercise increases gradually from 0% of the ‘‘basic” ATP usage at the onset of heavy-intensity exercise to 5% of the ‘‘basic” ATP usage after 6 min of this exercise. In trained muscle this (fitted within the model) increase of the ‘‘additional” ATP usage was smaller and was equal to 2.5%.

We had to fit a few parameter values (A_UT_, A_OX_, A_GL_, ‘‘additional” ATP usage) in order to reproduce well our experimental data. This was a minimum number of adjustable parameter values necessary to account for the measured effect of training within the computer model used. The model does not take into account explicitly the possible effect of H^+^, P_i_, ADP and other fatigue-related factors on (the efficiency of) such ATP-ases as actomyosin-ATPase, Ca^2+^-ATPase, Na^+^/K^+^-ATPase or Na^+^/Ca^2+^ exchanger. However, this effect is at least partly taken into account as the ‘‘additional” ATP usage. Of course, there are several other approximations or simplifications that always appear in theoretical models of complex real systems.

## Results

### Cardiorespiratory variables and plasma lactate concentration

The 20 weeks of endurance training resulted in no significant (~4%) increase in V’O_2peak_ (*P* = 0.12) and no significant changes in maximal minute ventilation (V’_Emax_), maximal heart rate (HR_max_) and maximal plasma lactate concentration ([La^-^]_pl max_) (see Table 1). The carbon dioxide production (V’CO_2_) and respiratory exchange ratio (RER) reached at the peak power output after training were significantly higher than before training (see Table 1).

**Table 1:** Values of peak power output, selected cardio-respiratory variables and plasma lactate concentration obtained at exhaustion during the incremental exercise test performed before and after 20 weeks of endurance training.

Variables	Before training		After training		*P value*
	Mean ± SD	95% CI of mean	Mean ± SD	95% CI of mean	
V’O_2 peak_ (mL · min^-1^)	3198 ± 458	2907–3489	3324 ± 325	3117–3530	*0*.*12*
V’_E max_ (L · min^-1^)	92.7 ± 16.4	82.3–103.1	101.5 ± 18.9	89.5–113.5	*0*.*09*
V’CO_2 max_ (mL · min^-1^)	3582 ± 497	3266–3897	3894 ± 477	3591–4197	*0*.*02*
RER	1.12 ± 0.03	1.10–1.14	1.17 ± 0.04	1.14–1.20	*0*.*02*
HR_max_ (bt · min^-1^)	187.2 ± 8.0	182.1–192.2	188.2 ± 8.5	182.8–193.5	*0*.*53*
[La^-^]_pl_ (mmol · L^-1^)*	8.1 ± 1.8	6.9–9.3	8.5 ± 2.0	7.2–9.9	*0*.*66*

Data are given as mean ± SD and the 95% CI of mean, n = 12.

* Maximal plasma lactate concentrations data are shown for eleven subjects. (*P* value was calculated using non-parametric Wilcoxon signed-rank test).

**Fig 3:**
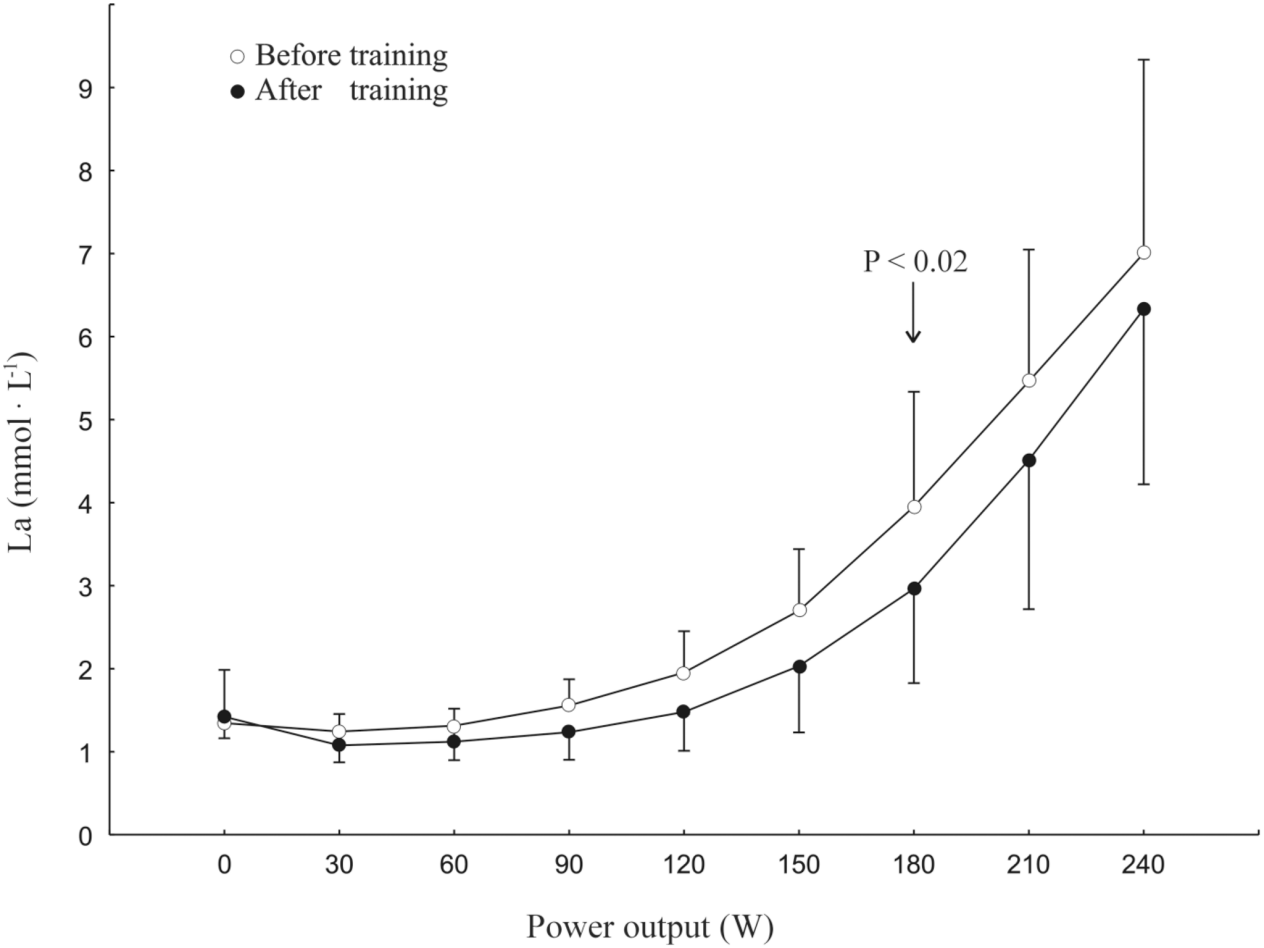
Plasma lactate concentration [La^-^]_pl_ during an incremental exercise test before (white circles) and after (black circles) 20 weeks of endurance training. The power output was increased by 30 W every 3 minutes up to 240 W. Note the right-ward shift of the lactate curve after training and especially the difference (*P* < 0.02 Wilcoxon signed rank test) between [La^-^]_pl_ at 180 W i.e. near to the power output at which the V’O_2_ on-kinetics has been determined, before and after the training. Data are given as means ± SD for 11 subjects.

Training resulted in a right-ward shift of the lactate *vs*. power output curve during the incremental exercise test (see Fig 3).

### Physical performance

Training resulted in a significant (*P* = 0.01) increase (by ~19%) of the power output at the LT (see Table 2). Also the power output at the V’O_2peak_ was significantly (*P* = 0.003) higher (by ~14%) after training (see Table 2).

**Table 2:** Physical performance (cycling and running) before and after the 20 weeks of cycling endurance training.

Cycling performance	Before training		After training		*P value*
	Mean ± SD	95% CI of mean	Mean ± SD	95% CI of mean	
PO at LT (W)	120.0 ± 28.6	101.8–138.2	142.5 ± 25.0	126.0–159.0	*0*.*01*
PO at V’O_2peak_ (W)	231.7 ± 35.1	209.3–254.0	263.4 ± 33.6	242.0–284.8	*0*.*003*
**Running performance**					
v at 85% HR_max_ (m · s^-1^)	2.83 ± 0.33	2.62–3.04	3.00 ± 0.31	2.80–3.19	*0*.*006*
v during 1,500-m run (m · s^-1^)	4.16 ± 0.33	3.96–4.37	4.37 ± 0.44	4.09–4.65	*0*.*001*

Data are given as mean ± SD and the 95% CI of mean, n = 12. (*P* value was calculated using non-parametric Wilcoxon signed-rank test).

Running performance, estimated by the running velocity at 85% of HR_max_ was ~6% higher (*P* = 0.006) after training (see Table 2). Moreover, the mean running velocity during the 1,500-m run was ~5% higher after training (*P* = 0.001) (see Table 2).

### Pulmonary V’O_2_ on-kinetics

**Fig 4:**
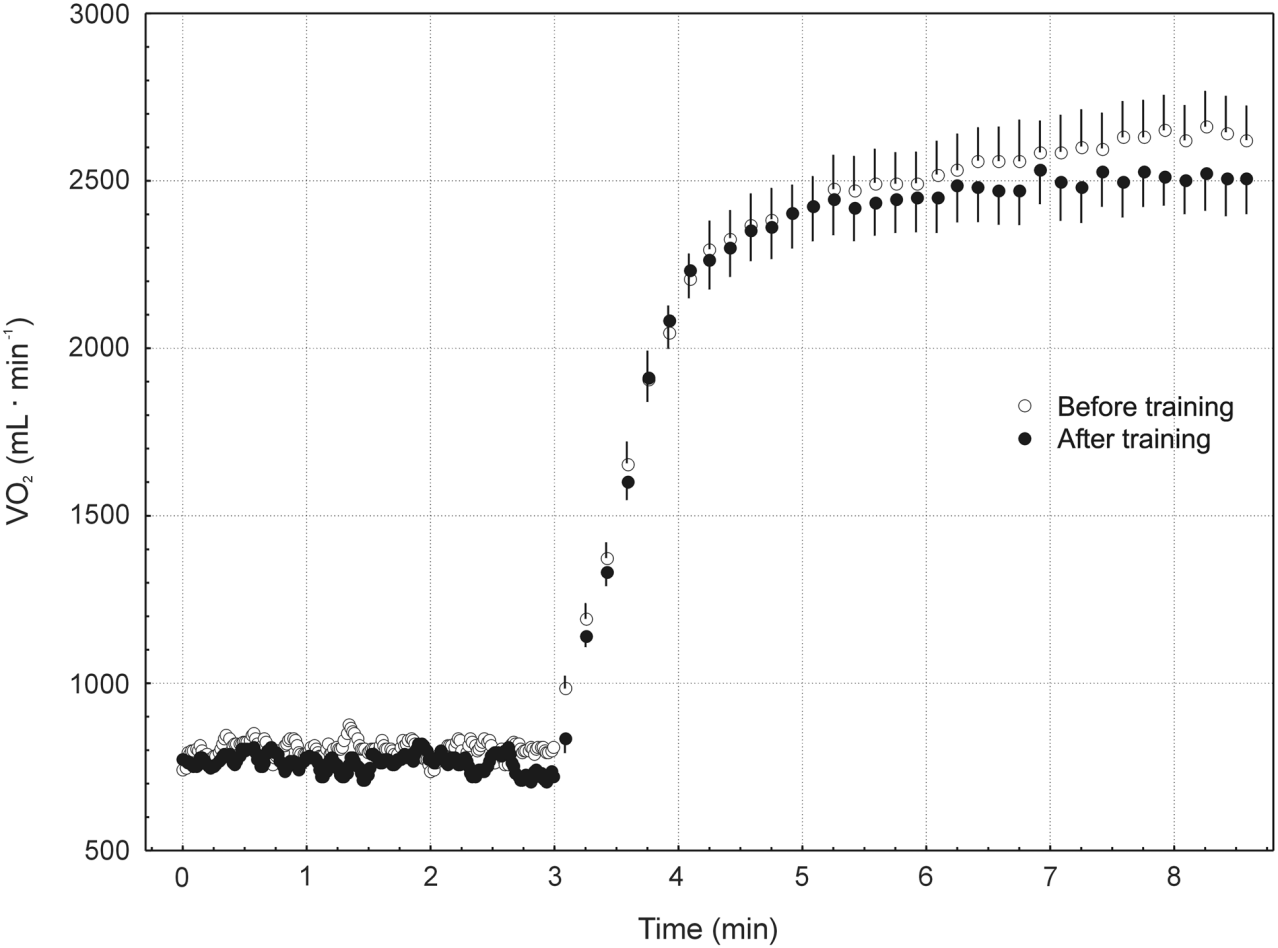
Mean (± SD) values of pulmonary oxygen uptake (V’O_2_) for 12 subjects during baseline and during heavy-intensity transition. Note the training-induced attenuation of the slow component of the V’O_2_ on-kinetics during high-intensity cycling.

Mean (± SD) V’O_2_ values, averaged over 10 seconds, before and during a constant power output cycling exercise performed before and after training are presented in Fig 4.

Training resulted in a significant (*P* = 0.027) decrease (by ~5%) of the baseline (20 W) V’O_2_ (see Table 3). This was accompanied by a significant (*P* = 0.007) shortening (by ~18%) of the phase II τ of the V’O_2_ on-kinetics. No effect of training on the phase II gain and absolute amplitude was found. A significant (*P* = 0.005) decrease (by 49%) of the magnitude of the slow component was found. Moreover, a significant (*P* = 0.0005) decrease (by ~5%) of the end-exercise V’O_2_ was observed after training. Moreover, a significant decrease (*P* = 0.005) in the end-exercise gain was found after training (as shown in Table 3).

**Table 3:** Oxygen uptake on-kinetics during high-intensity cycling exercise, before and after 20 weeks of training.

	Before training		After training		*P value*
	Mean ± SD	95% CI of mean	Mean ± SD	95% CI of mean	
Baseline O_2_ uptake (mL · min^-1^)	809 ± 97	747–871	766 ± 70	722–810	*0*.*027*
Phase II time constant (s)	30.1 ± 5.87	26.3–33.8	25.4 ± 5.10	22.1–28.6	*0*.*007*
Phase II amplitude (mL · min^-1^)	1668 ± 281	1490–1847	1661 ± 328	1452–1869	*0*.*97*
Phase II gain (mL · min^-1^ · W^-1^)	10.8 ± 0.6	10.4–11.1	10.7 ± 0.9	10.1–11.3	*1*.*0*
O_2_ uptake slow component time delay (s)	160 ± 35	137–182	93 ± 74	46–140	*0*.*012*
O_2_ uptake slow component (mL · min^-1^)	186 ± 88	130–242	94 ± 90	36–151	*0*.*005*
Relative contribution of slow component to net increase in V’O_2_ at end-exercise (%)	9.9 ± 3.8	7.5–12.3	5.3 ± 5.3	1.9–8.7	*0*.*012*
End-exercise O_2_ uptake at 6^th^ minute (mL · min^-1^)	2664 ± 369	2430–2899	2520 ± 362	2290–2750	*0*.*0005*
% peak O_2_ uptake	83.5 ± 5.2	80.2–86.8	75.7 ± 6.5	71.6–79.8	*0*.*002*
End-exercise gain (mL · min^-1^ · W^-1^)	11.9 ± 0.4	11.7–12.2	11.3 ± 0.7	10.9–11.7	*0*.*005*
Power output (W)	176.1 ± 30.2	156.9–195.3	176.1 ± 30.2	156.9–195.3	*1*.*0*
Gain power output (W)	156.1 ± 30.2	136.9–175.3	156.1 ± 30.2	136.9–175.3	*1*.*0*

Data are given as mean ± SD and the 95% CI of mean, n = 12. (*P* value was calculated using non-parametric Wilcoxon signed-rank test).

### Muscle proteins

The results of the maximal COX activity, citrate synthase content, MyHC composition (electrophoresis) and mtDNA copy number in relation to V’O_2_ on-kinetics during moderate-intensity cycling in the same group of subjects (n = 10) has been presented in our recent paper (for details see Ref. [28]). No significant changes in myosin heavy chain composition determined by SDS-PAGE has been found (*P* = 0.13) after training [28]). Additionally, in this study, no significant difference in MyHC2 content (Western immunoblotting) after the training was found (*P* = 0.90). Maximal COX activity showed a tendency to be slightly higher (by ~5%) after training (*P* = 0.08). A significant increase (*P* = 0.03) in mitochondrial DNA copy number in relation to nuclear DNA copy number was found after the training in relation to pre-training status [28]. Moreover, in the present study we have found no significant changes (*P* > 0.05) in the mRNA levels of mitofusin 1 (MFN1), mitofusin 2 (MFN2) and optic atrophy 1 (OPA1). This training resulted in a significant (*P* = 0.02) decrease (by ~22%) in SERCA2 content, whereas no significant changes in SERCA1 content were found (*P* = 0.73).

### Muscle capillarization

No changes in the mRNA levels of vascular endothelial growth factor A (VEGFA) (*P* = 0.24), hypoxia-inducible factor-1 (HIF1) (*P* = 0.21) and vascular endothelial growth factor receptor 1 (VEGFR1) (*P* = 0.12) were observed. However, a significant increase (~40%) in vascular endothelial growth factor receptor 2 (VEGFR2) mRNA level was found (*P* = 0.02). Moreover, this increase in VEGFR2 expression was accompanied by a clear tendency (*P* = 0.06) to higher capillary to fiber ratio (1.65 ± 0.11 vs. 1.98 ± 0.44 1· mm^2^, respectively, before and after training), where 95% CI of mean of this ratio amounted to 1.57–1.72 1· mm^2^ before training and to 1.67–2.29 1· mm^2^ after training.

### Computer simulations

**Fig 5:**
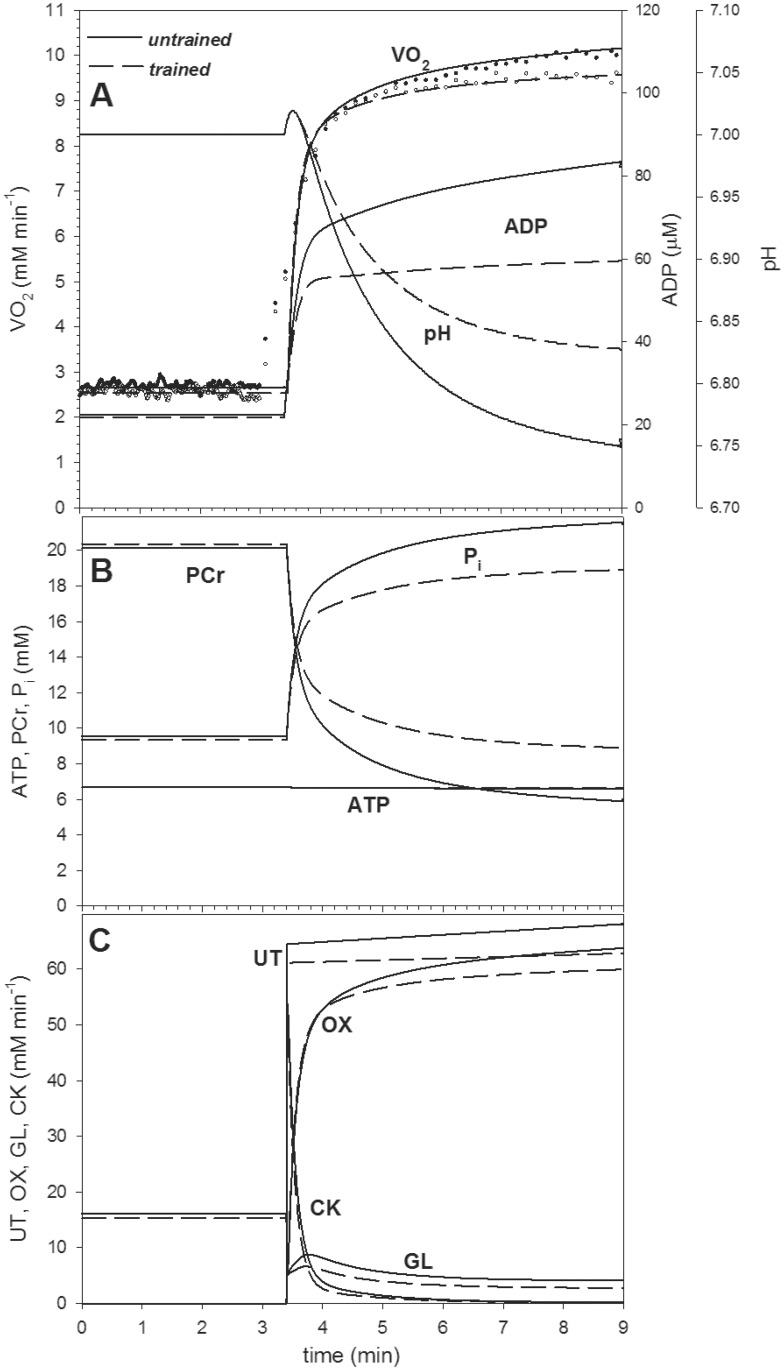
Experimental V’O_2_ and simulated muscle V’O_2_, metabolite concentrations and ATP usage/supply fluxes during low-intensity (baseline) and high-intensity cycling exercise in untrained and trained muscle. (A) Experimental and simulated V’O_2_, simulated ADP and pH. (B) Simulated PCr, P_i_ and ATP. (C) Simulated ATP usage (UT), ATP supply by OXPHOS (OX), ATP supply by anaerobic glycolysis (GL), ATP supply by creatine kinase (CK). Experimental baseline-heavy-intensity exercise transition: after 3 min. Simulated baseline-heavy-intensity exercise transition: after 3.4 min (the delay by 24 s corresponds to the cardio-dynamic phase of the pulmonary V’O_2_ on-kinetics). The muscle V’O_2_ is calculated based on the assumption that during baseline-intensity exercise (20 W) muscle V’O_2_ constitutes ~75% and during heavy-intensity exercise ~85% of the pulmonary V’O_2_ (see Ref. [40]).

The simulated time courses of V’O_2_ during baseline-heavy-intensity-exercise transition (for fitted A_UT_, A_OX_ and ‘‘additional” ATP usage values) agree well with the experimental ones (see Fig 5A). Computer simulations reproduce well the experimental finding that the training caused a decrease in baseline V’O_2_ measured during cycling at 20 W and, especially, in V’O_2_ during heavy-intensity exercise. The latter was caused by the assumed/fitted decrease of the ATP cost of exercise (by 5.5%) and, especially, by the postulated decrease in the ‘‘additional” ATP usage after 6 min of exercise from 5.0% to 2.5% of the ‘‘basic” ATP usage (without the ‘‘additional” ATP usage). The decreased additional ATP usage, being in our opinion one of the two main factors responsible for the slow component of the V’O_2_ on-kinetics [29], leads to a decrease of the magnitude of the slow component. This is demonstrated in Fig 5A. Fig 5A–5C presents also simulated time courses of other variables (metabolite concentrations and ATP fluxes) during the on-transition for untrained and trained muscle. Computer simulations suggest that physical training significantly improved muscle metabolite and pH stability during the on-transition: the increases in ADP and P_i_ and decreases in PCr and pH were indeed significantly smaller in trained muscle (Fig 5A and 5B). ATP usage was lower after training during low-intensity exercise (20 W) and, especially, heavy-intensity exercise, according to lower ‘‘basic” and ‘‘additional” ATP usage, as described in Methods. Consequently, ATP supply by OXPHOS and anaerobic glycolysis were also lower. The training-induced decrease in anaerobic glycolysis, which was attributable to ESA intensification (causing a smaller increase in ADP that indirectly activates glycolysis) and to lower direct activation of glycolysis at the onset of exercise (A_GL_ = 8.19 times instead of 8.41 times), led to a smaller cytosol acidification and thus contributed to the decreased magnitude of the slow component of the V’O_2_ on-kinetics (through smaller progressive glycolysis inhibition in the course of heavy-intensity exercise). While the training-induced decrease in the ATP/P.O. ratio decreased gain (pulmonary V’O_2_/P.O. ratio) and the phase II amplitude (because the absolute ‘‘basal” ATP usage during main exercise was much higher than ATP usage during prior exercise, 5.5% of the former is much greater than 5.5% of the latter), the training-induced decrease in the anaerobic glycolysis intensity had the opposite effect, and the phase II amplitude remained approximately constant. Additionally, an intensification of ESA itself improved the metabolite stability [39]. A comparison of the fluxes of ATP usage and ATP supply by anaerobic glycolysis (Fig 5C) suggests that in the present study the gradual inhibition of the latter flux by accumulating protons contributed more to the slow component than the ‘‘additional” ATP usage, especially after training. However, this conclusion applies only to the first 6 min after the onset of exercise. As the glycolysis-inhibition-related part of the slow component declines with time, while the ‘‘additional”-ATP-usage-related part can continue to increase linearly with time (depending on whether P.O. is below or above the critical power, CP), the slow component size will increase in time and after, say, 15 min of exercise the latter would predominate.

## Discussion

In the present study we have found that a prolonged (20 weeks) endurance training program, composed mainly of moderate-intensity exercise, had no effect on the V’O_2peak_ but accelerated pulmonary V’O_2_ on-kinetics during heavy-intensity constant power output exercise, shifted the blood lactate vs. power output curve to the right, attenuated the magnitude of the slow component of pulmonary V’O_2_ on-kinetics and reduced the end-exercise gain of the V’O_2_ response. This was accompanied by an increase of cycling endurance performance, as judged by an increase of the power output at V’O_2peak_, as well as by an improved running performance (increases in running velocity at 85% HR_max_ and in running velocity during all-out track run over the distance of 1,500-m). Therefore, our study indicates that prolonged moderate-intensity endurance training, although being insufficient to increase V’O_2peak_, significantly enhanced endurance performance in humans, mainly by a reduction in the development of muscle inefficiency, as judged by an attenuation of the slow component of V’O_2_ on-kinetics and an increase of the power output at V’O_2peak_.

It was postulated recently [29] that the main mechanisms underlying the slow component of the V’O_2_ on-kinetics in skeletal muscle are a gradual inhibition of the ATP supply by anaerobic glycolysis by accumulating protons (together with a slow decay of ATP supply by CK) and an ‘‘additional” ATP usage continuously increasing during exercise. Both mechanisms elevate the potential mismatch between ATP supply and demand. Namely, the inhibition by accumulating protons of the glycolytic ATP production lowers the total ATP supply, while the gradually increasing ‘‘additional” ATP usage rises the total ATP demand. As a result, additional oxidative ATP supply must be recruited in order to match ATP demand, which is associated with progressively increasing V’O_2_ (the slow component of the V’O_2_ on-kinetics).

In the present study, we have demonstrated, using a theoretical model of the skeletal muscle cell bioenergetic system, that a training-induced increase in ESA intensity leads to a decrease in the slow component through diminishing of the former mechanism intensity. This is because intensified ESA causes an enhanced oxidative ATP supply, lowers the increase in ADP during exercise and consequently decreases the (anaerobic) glycolytic flux and ATP supply from this source, and thus decreases cytosol acidification. Consequently, the fall in the glycolytic flux during exercise is smaller, which, together with a decrease in the ‘‘additional” ATP usage during exercise, decreases the slow component of the V’O_2_ on-kinetics (see Fig 5A–5C).

### The effect of training on cycling and running performance

The training program had no effect on the V’O_2peak_, but resulted in a significant enhancement of maximal cycling and running performance (see Table 2). Namely, the duration of the maximal incremental exercise test increased by ~12%. This was associated with an increase in peak power output at V’O_2peak_ by ~30 W i.e. by ~14% (*P* = 0.003). Our finding is in agreement with the previous study by Majerczak et al. [13], showing an increase in the power generating capabilities at V’O_2max_ after 5 weeks of moderate-intensity endurance training. The observed training-induced enhancement of power generating capabilities at a given V’O_2_ indicates that the moderate-intensity endurance training is potent to increase power generating capabilities at V’O_2 peak_ even in presents of unchanged V’O_2peak_. This seems to be especially important in case of people undertaking training of moderate intensity—known to be beneficial for enhancement of health status but less effective in an enhancement of V’O_2max_ than the high intensity training.

The reported increase in power generating capabilities at V’O_2peak_ can be explained by the training-induced improvement of muscle metabolic stability (see Fig 5A and 5B) and by the decreased O_2_ cost of cycling at high power outputs, as reflected by decreased slow component of the V’O_2_ on-kinetics (see Fig 2 and Table 3). One could speculate that this effect could be caused by an improvement of cycling technique during training (learning effect). However, in the present study the applied endurance training regimen (cycling) enhanced also running performance, both at submaximal as well as at maximal intensities (see Table 2). This strongly suggests that the attenuation of the slow component of the pulmonary V’O_2_ on-kinetics during cycling observed in the present study was mainly attributable to the training-induced enhancement of muscle metabolic homeostasis during exercise (increased ESA intensity and decreased ‘‘additional” ATP usage, see Fig 5 and discussion above). However, a direct comparison of those two kinds of movements requires some caution, since the “muscle working conditions” during running and cycling differs significantly e.g. in terms of tension development and its impact on muscle blood flow. Nevertheless, the enhancement of running performance after cycling training observed in the present study supports our suggestion that the observed attenuation of the magnitude of the slow component of the V’O_2_ kinetics during cycling originated mainly not from the learning effect but from the training-induced enhancement of muscle metabolic homeostasis during exercise.

### The effect of training on pulmonary V’O_2_ on-kinetics

In the present study training resulted in a significant (by ~16%) shortening of the τ_p_ of the V’O_2_ on-kinetics. This effect is similar to those obtained by a few weeks of training, as reported by others [41]. Such an acceleration has physiological significance [2,42,43]. First of all, a shortening of the τ_p_, in the presence of an unchanged amplitude of the primary component of the V’O_2_ on-kinetics decreases the size of the O_2_ deficit [2]. Moreover, the acceleration of pulmonary and (presumably) muscle [44] V’O_2_ on-kinetics suggests an enhanced muscle metabolic stability [21,28,39,42]. Namely, we have previously shown [39] that at a given ATP demand and under conditions where the CK reaction works near the thermodynamic equilibrium, the half-transition time *t*_0.5_ (and thus also τ_p_) of muscle V’O_2_ on-kinetics is determined by the amount of PCr that has to be converted into creatine (Cr) during the rest-to-work transition. Consequently, a linear relationship between half-transition time of muscle V’O_2_ on-kinetics and the ΔPCr during rest to work transition has been demonstrated (see Ref. [39]–Fig 4 therein). This concept is in agreement with experimental data showing a significant positive correlation between the magnitude of decrease in muscle PCr concentration during exercise and the rate of pulmonary V’O_2_ on-kinetics in humans [19,45]. Therefore, the observed training-induced acceleration of muscle V’O_2_ on-kinetics can be considered as an indirect marker of an enhanced muscle metabolic stability, as described by lesser changes in muscle PCr, P_i_, ADP_free_, H^+^, AMP, IMP, ΔG_ATP_, etc. during rest to work transition [21,28,39,42,43]. In this respect, the attenuation of the computer-simulated increases in muscle P_i_, ADP_free_, and lesser decreases in PCr and pH, as well as the lower anaerobic glycolytic flux after training (Fig 5A–5C) appear noteworthy. Moreover, in the present study we have also found a significant right-ward shift of the plasma lactate concentration curve during the incremental test (see Fig 3), resulting in an ~19% increase of the power output at the LT. This additionally supports our hypothesis that the applied training enhanced muscle metabolic stability during high-intensity exercise and decreased the ATP supply by anaerobic glycolysis.

### MyHC content and the slow component of the pulmonary V’O_2_ on-kinetics

In the present study we have observed a significant decrease (by ~50%) of the magnitude of the slow component of the V’O_2_ on-kinetics (see Fig 4 and Table 3). Similar effects, occurring after a few weeks of varied kinds of training has been reported by others [10,11,12]. The mechanism by which training attenuates the slow component remains unclear (for review see Ref. [41]). It has been reported that the relative amplitude of the slow component of V’O_2_ kinetics during exercise of heavy- and severe-intensity was significantly and negatively correlated with the percentage of type I fibers [46,47]. It has been suggested that the training-induced attenuation of the slow component could be due to a lesser recruitment of type II muscle fibers [41]. On the other hand Zoladz et al. [9], using an electrically stimulated isolated dog gastrocnemius in situ preparation, reported a clear V’O_2_ slow component-like response, i.e. a progressive increase of V’O_2_: force ratio in the absence of a progressive recruitment of muscle fibers (see Ref. [9], Fig 4 therein). These authors concluded that not recruitment of type II muscle fibres *per se*, but rather the consequence of their recruitment, i.e. disturbances in muscle metabolic stability (i.e. an increase in ADP_free_, P_i_, H^+^, IMP, AMP, NH_3_ concentrations, decrease in muscle PCr concentration and ΔG_ATP_) in already working muscle fibers would be responsible for the slow component [9]. This could be due to the metabolites induced decrease of mechanical efficiency and muscle fatigue of the already recruited muscle fibers [3,9,48,49], leading to an increase of the ATP cost of force and power generation (“additional ATP usage”). Namely, it has been postulated that the slow component of the V’O_2_ on-kinetics is a consequence of the muscle fatigue-induced decrease of muscle efficiency [9]. This concept recently received some additional experimental supports [13,42,50,51]. In the present study we have found a pronounced attenuation of the slow component of the V’O_2_ on-kinetics in the presence of no changes in the proportion of MyHC in the vastus lateralis muscle, which suggest that the training-induced decrease of the slow component in humans does not require changes in MyHC composition. Interestingly, in the present study we found a significant (*P* = 0.02) decrease in SERCA2 content (by 22%) following training. This could contribute to an enhancement of type I muscle fibers efficiency and to a decrease in the O_2_ cost of power generated by these muscle fibers [13,52] (in particular: of the ATP/P.O. ratio). The role of type I muscle fibers in the origin of the slow component of the V’O_2_ on-kinetics is not obvious, but there is a growing body of evidence that their fatigue-induced decrease of mechanical efficiency can contribute to the slow component [9,53].

### Muscle acidosis and the slow component of V’O_2_ kinetics

It is well established that the slow component of the V’O_2_ on-kinetics (or the slow-component-like response, [9]) is associated with metabolic acidosis. Namely, the slow component is normally present during exercise exceeding the lactate threshold [4,5] and becomes progressively greater after exceeding the “critical power” [1,3,6,7]. Accordingly, it has been reported that the amplitude of the slow component is linearly related to the level of lactate accumulated in blood [7,54,55]. Moreover, it has been shown by Casaburi et al. [11] that the training-induced decrease in blood lactate concentration is correlated with the attenuation of the amplitude of the slow component. On the other hand, it was reported that pharmacologically induced enhancement of blood lactate concentration had no effect on the magnitude of V’O_2_ slow component in humans (for discussion of this point see Ref. [29]). Moreover, as demonstrated by Poole et al. [56], direct infusion of L-(+)-lactate, increasing lactate concentration in blood and in contracting muscles had no significant effect on the amplitude of the V’O_2_ slow component in dog gastrocnemius muscle. On the other hand, pre-exercise induced metabolic acidosis, obtained by ingestion of NH_4_Cl, significantly increased the magnitude of the slow component of V’O_2_ on-kinetics in humans [57]. These findings strongly suggest that muscle acidosis, not lactatemia, is involved in the appearance of the slow component of the V’O_2_ on-kinetics. Although H^+^ accumulation in muscle can affect its function by decreasing its efficiency and inducing fatigue [3], muscle acidosis developed during exercise is normally accompanied by greater disturbances in muscle metabolic stability, including an increase in ADP, P_i_, and a decrease in PCr concentration and ΔG_ATP_, which can also affect muscle efficiency [3,9,48,49]. Decreased efficiency of the recruited muscle fibers [9] would lead to additional ATP usage, which could increase gradually during a high-intensity exercise, resulting in a slow component of the V’O_2_ on-kinetics. It has been demonstrated by a theoretical model of skeletal muscle bioenergetics, including anaerobic glycolysis, that the inhibition of ATP production by anaerobic glycolysis induced by progressive cytosol acidification (together with a slow decrease in ATP supply by creatine kinase) as well as a gradual increase in ‘‘additional” ATP usage during high intensity exercise, would lead to development of slow component of the V’O_2_ on-kinetics in order to maintain the required ATP supply [29]. The training-induced intensification of ESA and decrease in the ‘‘additional” ATP usage would diminish ADP concentration during exercise, and thus would inhibit (anaerobic) glycolysis. This in turn would lead to a smaller cytosol acidification during exercise and consequently to a smaller decrease in ATP supply by anaerobic glycolysis in the course of exercise. Altogether, the training-induced enhancement of muscle metabolic and pH stability, caused by ESA intensification and ‘‘additional” ATP usage decrease, can lead to a decreased magnitude of the V’O_2_ slow component via a decrease in the potential mismatch between ATP production and consumption. Lower cytosol acidification causes lower decrease of ATP supply by anaerobic glycolysis during exercise, while lower ‘‘additional” ATP usages causes lower increase in the total ATP consumption during exercise.

Generally, the sequence of events is as follows. 1. ESA intensification and ‘‘additional” ATP usage decrease cause a smaller increase in ADP and P_i_ during exercise; 2. (Anaerobic) glycolysis is stimulated to a smaller extent; 3. Cytosol acidification is smaller; 4. Decrease in the glycolytic ATP supply caused by accumulating protons during exercise is smaller; 5. Increase in oxidative ATP supply in order to match ATP usage (which is additionally decreased due to a decrease in ‘‘additional” ATP usage) is smaller; 6. Increase in V’O_2_ is smaller, which means a smaller slow component.

Therefore, the regulation of (anaerobic) glycolysis is self-limiting. An increase/decrease in (anaerobic) glycolysis intensity at the onset of exercise causes an increase/decrease in cytosol acidification during exercise. This in turn increases/decreases the progressive glycolysis inhibition during exercise. In short, the more glycolysis is activated at the onset of exercise, the more it is afterwards inhibited in the course of exercise.

It must be emphasized that a rather low magnitude of the slow component V’O_2_ on-kinetics was encountered in the present study, compared to other experimental studies [4,7]. Probably this was mainly caused by a relatively low ‘‘additional” ATP usage. The ‘‘additional” ATP usage of 2.5% (after training) or 5% (before training) after 6 min of exercise was assumed in the present study in order to match experimental data, while this value was 20% in our previous theoretical study [29]. The relatively small magnitude of the measured slow component of the V'O_2_ on-kinetics and of simulated ‘‘additional” ATP usage in the present study was due to the fact that we have applied a bout of exercise corresponding to 50% Δ (for detail see the Methods sections), which is commonly used to “scale”the exercise intensity when studying the V’O_2_ kinetics in humans [10,11,41,46]. Nevertheless, this exercise intensity by definition is far below that one (near V’O_2max_) which could generate maximal magnitude of the slow component of V’O_2_ on-kinetics (see e.g. Ref. [4,7]).

During the 6 min of exercise applied in the present study the relative increase in the ATP usage flux (‘‘additional” ATP usage) is less than the relative decrease in the ATP supply flux by anaerobic glycolysis (Fig 5C). This fact suggests that the gradual inhibition of the latter by accumulating protons contributed more to the slow component than the ‘‘additional” ATP usage, especially after training. However, in exercises of longer duration times, say 15 min, this conclusion would be no longer valid. As the glycolysis-inhibition-related part of the slow component declines with time, while the ‘‘additional”-ATP-usage-related part can continue to increase linearly with time (depending on whether P.O. is below or above CP), the latter would predominate.

### Each step activation and V’O_2_ on-kinetics

It has been proposed in previous theoretical studies that the two main mechanisms possibly responsible for the training-induced acceleration of muscle V’O_2_ on-kinetics are an increased OXPHOS activity related to mitochondria biogenesis and ESA intensification [21,39]. In our recent study concerning moderate-intensity exercise [28], involving the same subjects and training protocol of the present study, we concluded, after analyzing the changes in markers of mitochondrial biogenesis (mtDNA copies, CS amount and activity, COX activity) that little or no training-induced increase in OXPHOS activity took place. Moreover, in the present study we have found no significant changes in other markers of mitochondrial biogenesis such as the mRNA level of mitofusin 1 (MFN1), mitofusin 2 (MFN2) and optic atrophy 1 (OPA1). Therefore, we postulate that, also for high-intensity exercise, the observed training-induced acceleration of the pulmonary V’O_2_ on-kinetics and the related improvement of metabolite homeostasis were mainly or exclusively due to ESA intensification.

### Oxygen delivery and V’O_2_ on-kinetics

Some impact of the training-induced enhancement of muscle blood flow during exercise on the V’O_2_ on-kinetics cannot be excluded. It has been recently pointed out by Murias et al. [58] that the V’O_2_ on-kinetics can be limited, at least in part and in some experimental conditions, by O_2_ delivery and/or the intramuscular matching O_2_ delivery and O_2_ utilization as postulated by Koga et al. [59]. Although during exercise of moderate-intensity such scenario seems unlikely (for review see Ref. [60]), during heavy-intensity exercise a limitation of O_2_ supply could lead to a decrease in the mitochondrial O_2_ level, to an impairment of oxidative metabolism and to a slower VO_2_ kinetics (as well as to the appearance of the slow component). Inadequate O_2_ availability could affect muscle metabolite concentrations, including an increase in ADP and P_i_ as well as a decrease in PCr [61,62,63] before limiting V’O_2max_ [64]. Such disturbances in metabolic stability could contribute to a decrease in muscle efficiency and an increase of ‘‘additional” ATP usage. In the present study we did not directly determine the effects of training on O_2_ delivery during high-intensity exercise. However, we evaluated the effects of training on the expression of some genes related to muscle capillarization in the vastus lateralis. Namely, we found a significant increase (by ~40%) in vascular endothelial growth factor receptor 2 (VEGFR2) mRNA levels after completing the training programme. Moreover, a tendency (*P* = 0.06) towards higher capillary-to-fiber ratio after the training was found. Therefore, we conclude that the applied moderate-intensity training could attenuate the magnitude of the slow component at least in part, by an enhancement of muscle metabolic stability caused by an increased O_2_ delivery.

### Study Limitations

Another factor which could be affected by training and which could decrease the magnitude of the slow component would be an increase in the coupling of OXPHOS [65,66]; this factor was not evaluated in the present study. Nevertheless, we estimated in a recent study [29] (based on the available data, see therein) that the contribution of proton leak to V’O_2_ during heavy exercise is rather small. However, in view of the very recent study [66] the potential effect of changes in efficiency of oxidative phosphorylation (P/O) during exercise on the magnitude of the slow component of V’O_2_ kinetics in untrained and in trained status requires further experiments. In the presents study we also do not consider the potential effect of the training-induced increase in fatty acid oxidation at the cost of a decrease in carbohydrate oxidation by working muscles [67] that could slightly diminish the ATP/O_2_ ratio, as this effect would be small and act in the opposite direction than the training-induced changes found in our study. We should also consider that any computer model is only an approximation of the complex reality (compare the subsection ‘‘Computer simulations of muscle V’O_2_, metabolite changes and ATP fluxes during work transitions”). Therefore, the model used in the present study should be further verified by comparing its predictions with a broader set of system properties in different muscles and various experimental conditions, as it was done in a recent paper [68].

## Conclusions

A prolonged (20 weeks) endurance training program composed mainly of moderate-intensity exercise attenuated the slow component of V’O_2_ on-kinetics and enhanced maximal cycling and running performances. This was accompanied by no changes in MyHC composition or enhancement of OXPHOS activity in the trained quadriceps muscle, although a modest increase in muscle fiber capillarization was observed. On the basis of simulations carried out using a computer model of skeletal muscle bioenergetics published previously, we postulate that the acceleration of the V’O_2_ on-kinetics and the attenuation of the slow component were mainly caused by a training-induced enhancement of ESA (each-step activation) of OXPHOS and by a decrease in an ‘‘additional” ATP usage, progressively increasing during exercise. Both effects would reduce the possible mismatch between ATP supply and demand and would increase muscle metabolite stability. The consequent decrease in the ADP rise at the onset of exercise would diminish the (anaerobic) glycolytic flux and reduce cytosol acidification, leading to a smaller inhibition of ATP supply by anaerobic glycolysis in the course of exercise. Therefore, the stronger (anaerobic) glycolysis flux increase at the onset of exercise, the more pronounced advancing glycolysis inhibition in the course of exercise (self-limitation phenomenon).
